# Healthy Perturbation

**Published:** 1920-03-13

**Authors:** 


### THE PUBLIC WELL-BEING.

### Healthy Perturbation.

### Leeds Agitated by Military Health Report. Advantage of Local Discussion

The alarming report of the results of medical examinations under the National Service scheme from November 1917 to October 1918 should do a great deal to exercise the public mind in the direction of all possible steps to remedy the serious state of things which the report discloses. It has been widely discussed in all papers, and one particular advantage to be derived from it is where it deals specifically with particular areas. The result of this is pointedly demonstrated in the case of Leeds, and if we deal rather fully with that case it is because of the wider application involved. There were some rather severe strictures concerning the conditions in Leeds, with the consequence that there is to be found in the Leeds newspapers a very animated discussion amongst medical men and others as to the truth or otherwise of the deplorable conditions alleged by the report. The author of the report, Professor Keith, who has been interviewed by the *Yorkshire Evening Post*, offers the wise advice that energy should be expended, not in finding excuses for the state of things revealed, but in seeking a remedy. He tells Leeds frankly that there is something radically wrong with it, and that there are the gravest reasons why it should be looked into. The condition of the population of Leeds needed, he said, serious consideration as much as that of any city in the country, though he agreed there were worse cities than Leeds.

Some of the best comments from Leeds itself came from the medical officer of health, who said the facts must be faced, that there was a direct call to the city to rectify them, and that there was no longer any excuse that they did not know the truth. The most important feature in his statement in the *Yorkshire Post* was what was urged in these columns last week—namely, that we have got to get back to the beginning of things and concentrate on motherhood and all that it implies if any solid reformation is to be effected.

"If we are going to have fit men and women," he says, "we must have fit children, and to get fit children we must have fit mothers. In my opinion, everything depends upon the mother. A pound spent on the mother and her baby is worth £100 spent on the adult. In an industrial city like Leeds, there are a great many things which militate against a healthy condition of things. In the first place, a large number of girls—and girls are the potential mothers—spend the greater part of their young life in factories and workshops. Then there is the fact that certain callings in which girls are engaged are quite unsuitable to them in view of their future position as mothers. For example, work in the dusty trades—a very general thing among girls—and heavy manual work are both bad for girls. It is very difficult to say that any one of the trades included in those groups is worse than another.

"Then there is the lack of preparation for parenthood. Girls are not taught to prepare themselves to become mothers, nor are boys taught to prepare themselves to become fathers. That means that the mother has to get practically all her knowledge from experience after the birth of the child, and that while she is getting that experience the child is deteriorating. Then there are the general surroundings of the home. In Leeds, these are not good. I do not say they are worse than in any other town, but in order to get healthy parents you must have healthy homes. It is, of course, a combination of conditions which gives rise to unfitness. I do not think any one of these can be said to be the cause. I am perfectly convinced that Leeds is no worse than Sheffield or Manchester, or any of the large towns, in respect of any of the conditions I have mentioned. We have to go deeper if we want to get the truth."

The chairman of the Health Committee said everything was being done to improve the conditions and to make the population healthy, and, though he knew that back-to-back houses were at least not the best possible, at the same time he did not think they were as harmful as was sometimes supposed. However, they hoped that such houses would be rapidly superseded. With regard to the employment of women in unsuitable trades, the chairman said he was "dead against" married women being employed at all, except in looking after their homes and their children, and every girl should be instructed in the bringing up of children.

A fair point was made by a well-known Leeds consultant, Dr. W. H. Maxwell Telling. He said he had been practising in Leeds for twenty years, and his opinion of the physique of Leeds men, except for the Jews, of whom there were a large number in the city, was not in accordance with that of Professor Keith, whose deductions were based on evidence acquired at the most unfavourable time, and for that reason were fallacious. He contended that the vital statistics of Leeds compared favourably with other towns, and these, he declared, were the best indications of health conditions. But the death statistics for 1918, contained in the report of the Leeds medical officer, do not seem to give him great support. They show that out of fifteen leading cities in England, Leeds was twelfth, with a death-rate of 19.2 per thousand. The cities which were below her were Sheffield, Hull, and Nottingham. It is true that this was the highest rate recorded since 1900, and this circumstance was attributed to phenomenal epidemics of influenza, superimposed upon a severe outbreak of measles earlier in the year. At the same time, it must be borne in mind that the influenza epidemic was widespread throughout the country, and in Sheffield, for instance, whose death-rate was 20.5 for 1918, the influenza epidemic was intense. The conditions in Leeds, therefore, seem not to have been exceptional, and, at any rate so far as 1918 is concerned, Dr. Maxwell Telling's point does not look a particularly strong one in favour of Leeds. Of all the cities included in the number given, Birmingham was first with 15.2, Huddersfield second with 16.7, London, Bradford, Liverpool, Halifax were all just over 19, and Hull was worst, with 21.9. The average for England and Wales was 17.6.

So that, allowing for the natural bias of local patriotism, it cannot be very strongly concluded that Leeds has made good its protest. One significant fact is reported by the *Yorkshire Evening Post*. One of its representatives visited a school in a poorer district of South Leeds, and was told that out of several hundred scholars there, only one had a physique which came up to the normal standard. One teacher said the bodies of the children were not well developed, and he attributed this to the lack of nourishing food and the bad conditions in which they lived. The Leeds boys who visited other towns to play football, added the teacher, were not so big as their opponents, and they could not stay the pace like some of the others. The Yorkshire papers have done a great service to Leeds and, in fact, to the North of England in general, by taking up this serious question, and it will be a good thing if all other regional and local papers will devote the same careful attention to the problem raised.

